# Menstruation and injury occurrence; a four season observational study in elite female football players

**DOI:** 10.3389/fspor.2025.1665482

**Published:** 2025-12-16

**Authors:** E. Ferrer, N. Keay, L. Balagué-Dobón, A. Cáceres, P. Jarrin, G. Rodas, J. R. González

**Affiliations:** 1 FC Barcelona Medical Department (FIFA Medical Centre of Excellence) and Barça Innovation Hub, FC Barcelona, Barcelona, Spain; 2 Sports and Exercise Medicine Unit, Hospital Clinic and Sant Joan de Deu, Barcelona, Spain; 3 Universitat de Barcelona (UB), Barcelona, Spain; 4 Division of Medicine, University College London, London, United Kingdom; 5 Barcelona Institute for Global Health (ISGlobal), Barcelona, Spain; 6 CIBER in Epidemiology and Public Health (CIBERESP), Barcelona, Spain

**Keywords:** female football, hormones, menstrual cycle, musculoskeletal injuries, calendar based

## Abstract

**Background:**

The menstrual cycle has been hypothesized to influence injury risk in female athletes due to hormonal fluctuations affecting musculoskeletal, metabolic, and neuromuscular systems. However, methodological inconsistencies and variability in phase classification have resulted in conflicting evidence. The lack of biological samples forces researchers to estimate and speculate about the relationship between the menstrual cycle and injury risk. It is well established that each phase of the cycle is characterized by specific hormonal profiles with distinct physiological functions. Without direct measurement of these hormone levels, it is difficult to generate accurate and reliable data. The only phase in which hormonal status can be confidently inferred is menstruation, as this phase is defined by low concentrations of ovarian hormones. Understanding this relationship in elite female football players is crucial for optimizing training load, health management, recovery strategies, and injury prevention.

**Objective:**

To investigate the association between the early follicular phase (menstruation) and the incidence of musculoskeletal time-loss injuries in elite female football players across four competitive seasons.

**Study design:**

Descriptive epidemiological study.

**Methods:**

Menstrual cycle and injury data were prospectively collected from 33 elite female football players between the 2019/20 and 2022/23 seasons. Menstrual cycle was tracked using a calendar-based digital tool, and injuries were classified according to the OSICS-10 coding system. Injury incidence rates per 1,000 h of exposure were computed and compared between bleeding and non-bleeding phases. It should be noted that in this article, the terms bleeding, menstruation, phase 1, and early follicular phase are used interchangeably to refer to the same stage of the menstrual cycle.

**Results:**

A total of 852 menstrual cycles were analysed, during which 80 injuries were recorded. Of these, 18 (22.5%) occurred during matches and 62 (77.5%) during training. The most common injury types were muscle injuries (57.5%), ligament injuries (30%), and tendon injuries (12.5%). Injuries during the bleeding phase accounted for 13.7% (*n* = 11) of all cases. The overall injury incidence rate was 6.42 per 1,000 h (95% CI: 5.09–7.99), with an incidence of 5.46 per 1,000 h during the bleeding phase and 6.60 per 1,000 h during non-bleeding phases (*p* = 0.55). Although injury incidence was not significantly different between phases, the injury burden was substantially higher during bleeding (684 vs. 206 days lost per 1,000 h; *p* = 0.0027), indicating that injuries sustained during menstruation resulted in more severe consequences.

**Conclusions:**

This study provides evidence that injury incidence is similar during menstrual bleeding compared to non-bleeding days in elite female football players. However, injuries occurring during menstruation are associated with a significantly higher burden, suggesting an increased risk of more severe injuries during this phase, these findings highlight the importance of individualized menstrual tracking for injury prevention and athlete health management. Further research with precise hormonal monitoring is needed to confirm these observations and to inform training, recovery, and health strategies in female athletes.

## Introduction

In recent years, growing attention has been directed toward the influence of the menstrual cycle on female sports. Historically studies have attempted to classify injury data across the different menstrual phases, yet accurate phase identification demands biochemical confirmation due to the unique hormonal profiles that define each phase (1) The menstrual cycle is a key physiological process in women, its impact on performance, neuromuscular control, injury risk, and overall well-being in elite athletes remains a topic of ongoing investigation (2). The cycle is characterized by fluctuations in ovarian hormones, which influence various physiological systems, including musculoskeletal integrity, metabolism, and immune response (3, 4). Among elite female football players, these hormonal changes may alter injury susceptibility across different menstrual phases (5, 6). Notably, studies have suggested a higher incidence of injuries during ovulation (2), while other evidence highlights increased injury risks during the luteal (7) or early follicular phase (8). Due to the variation in findings, among other factors, and given the participation of women in elite sports, understanding the relationship between menstrual cycle phases and injury susceptibility is crucial for optimizing training regimens, health management, recovery strategies, and injury prevention protocols.

An important challenge in menstrual cycle research is the heterogeneity in phase division and methodology (9). Different divisions of the menstrual cycle describe the same biological process but with different terminology, some classic publications develop the studies following a two-phase classification, follicular and luteal (10) while others use a wider phase model including four phases (phase 1, phase 2, phase 3 and phase 4) based on hormones fluctuation (9). Regarding the methodology, heterogeneity is also observed, some studies rely on self-reported phases, others use biomarkers, questionnaires or others computational models to estimate hormonal fluctuations (11). For instance, a large-scale study in elite female futsal players, based on blood test and hormone determination, reported a higher prevalence of severe injuries during the follicular phase, particularly in the ovulatory window (5). Conversely, other research in track and field female athletes, whose results were obtained through questionnaire responses, suggests that the late luteal phase may negatively impact performance and increase injury susceptibility (12) or a calendar-based study, via app, that had an integrated algorithm that assumed the individual hormonal profile, concludes that injury risk was higher during luteal phase (7). These discrepancies highlight the need for precise tracking methodologies and individualized assessments to better understand the implications of hormonal fluctuations on athletic performance and injury risk. Hormonal cycles, however, largely vary within and between individuals, and therefore, estimating menstrual phases without direct measurements is considered unreliable (11). If only menstruation is tracked, the menstrual cycle should only be divided into menstruation, an early follicular phase characterized by bleeding, and non-bleeding days. As such, there is a need to accurately estimate the effect of the early follicular phase on injury in closely followed elite female athletes, minimizing differences in their training conditions, performance, and workload.

Beyond hormonal changes during the cycle, other physiological changes associated with menstruation can further influence an athlete's vulnerability to injuries. Endometrial bleeding, lasting 4–8 days per cycle, contributes to iron loss, with menstruating women losing approximately 1 mg of iron per day (13). Iron deficiency has been linked to reduced training load and endurance performance, potentially exacerbating fatigue and impairing recovery (14). Additionally, injury risk can be affected by changes in metabolic, cardiovascular, and symptomatology, which may influence muscle recovery, inflammation, and neuromuscular coordination (15). These systemic changes underscore the importance of estimating the associated injury risk by early follicular phase of the menstrual cycle, which is characterized by low hormone concentrations, in elite female athletes.

Given the existing body of research and the inconsistencies in phase classification, this study aims to provide a more comprehensive understanding of the relationship between the menstruation and injury occurrence in a group of elite football players. Understanding these patterns will in the future, inform evidence-based training, prevention and recovery strategies, ultimately enhancing player health and performance in elite women's football.

## Material and methods

### Participants

Thirty-three elite football players, from a single professional team competing in Spain's Liga F (former Liga Iberdrola) was monitored over four consecutive seasons from 2019/20 to 2022/23, a period during which they won two UEFA Women's Champions League (UWCL) titles. Players using combined forms of hormonal contraception, or amenorrhoeic were excluded. A total of 17 players were included in season 19/20, 20 players for 20/21 season, 18 players in season 21/22 and 22 players for the last season 22/23, highlighting that 11 of the players were included along all the four seasons. Informed consent was obtained before data was gathered, and participants were informed that they could withdraw from the study at any time.

### Injury diagnoses, type of injury and severity (return-to play)

Data collected in relation to injuries sustained during these four seasons were included in the study. This study followed consensus guidelines on the definitions and data collection procedures for football injury studies described by UEFA (16). Only lower limb musculoskeletal injuries sustained during training or matches were considered. If they caused the player to be absent from at least the following training session or match, they were classified as time-loss (TL) injuries. Injuries were classified using the OSICS_10 coding system (Orchard Sports Injury Classification System) and focus on muscle injuries were indicated by the code (code OSICS -M-), ligament injuries (OSICS -J-) and tendon injuries (OSICS -T-). All the diagnoses were made by the same team doctor, and the rehabilitation and return to play process, followed the FC.Barcelona muscle and tendon injury guides (17, 18).

To calculate injury severity, we based on a UEFA model (16) that determined according to the number of days from injury occurrence until the end of medical leave, ranging from mild (1–7 days), to moderate (8–28 days), and severe (>28 days).

### Quantification of the GPS data

GPS data were collected using the WIMU PROTM device (RealtrackSystems S.L., Almeria, Spain). The data collected were analysed using the SPROTM Software (version 927; RealtrackSystems, Almeria Spain), which exports the data in RAW format.

Exposure time was calculated based on the data collected by the WIMU (Wireless Inertial Measurement Unit), which provided detailed information on player activity and duration during training sessions and matches.

### Menstrual cycle evaluation

Four-season menstrual cycle data were self-reported via club-managed app (E-keep), following a calendar-based method. Players logged on the onset and cessation of menstrual bleeding. Only bleeding days (menstruation, also known as the early follicular phase or phase one “1”, were used for the phase classification). Non-bleeding phases were treated as a composite phase due to the absence of hormonal data. All data was supervised by a unique professional (team doctor) who oversaw the monthly data collection. In case of any data missing, the same professional would ask individually for it and would include it in the data set for posterior analysis.

### Statistical analyses

Injury incidence and burden were calculated using R software. Incidence rates were computed as the number of injuries per 1,000 h of exposure, and burden was expressed as the number of days lost per 1,000 h of exposure. Ninety-five percent confidence intervals (95% CI) for incidence and burden were calculated assuming a Poisson distribution, following the consensus methodological guidelines for sports injury surveillance (16, 19). Estimates were stratified by exposure type (training vs. match), side, injury category, severity and bleeding or not bleeding. Correlation among injuries (e.g., differences in each player's predisposition to sustain more or fewer injuries) was controlled by using a Poisson mixed-effects model (via the *glmer* function in the R package lme4), with player ID included as a random intercept. This approach accounts for intra-player correlation and allows estimation of incidence rates per 1,000 h of exposure, with confidence intervals and *p*-values derived from comparisons with a null model.

### Ethics approval and informed consent

The study was conducted according to the guidelines of the Declaration of Helsinki nd was approved by the local committee of Barça Innovation Hub and the Ethics Committee of Consell Català de l'Esport (code 012/CEICGC/2021).

## Results

### Menstrual cycle data

Thirty-three elite football players (mean age: 25.72 ± 4.27 years) were studied. A total of 852 menstrual cycles were recorded and analysed across four seasons (2019/2020–2022/2023). All cycles were recorded using the club's application, with the study supervisor overseeing data accuracy. Once all dates were incorporated into the database, the average length of the menstrual cycle was calculated. Additionally, the mean duration of bleeding and non-bleeding phases was determined. The players’ average menstrual cycle length was 31 days, indicating that the inter-cycle duration aligns with the clinically normal range of 21–35 days (10, 20, 21).

Acknowledging that the mean cycle length was 31 days, with an average of four bleeding days, non-bleeding phases comprised approximately 87% of the total cycle duration.

### Associations between injury incidence, days lost, and burden along bleeding and non-bleeding days

The aim of the study was to accurately assess injury incidence and burden in the two menstrual cycle phases that can only be determined when no hormonal assays are available, namely bleeding and non-bleeding. These phases were cross-referenced with injury data, categorized by bleeding and non-bleeding, match or training, type, days loss and laterality (Table 1).

**Table 1:** Cross-referenced data between bleeding and non-bleeding phases with injury data.

Injury data	Category	*N*	N° days lost	Median days lost	Injury incidence	Injury burden
Bleeding Status						
	Bleeding	11 (13.7)	1,379	53	5.36	684
	Non-bleeding	69 (86.2)	2,151	17	6.48	206
	*p*-value				0.5519	0.0027
Exposure Type						
	Match	18 (22.5)	784	23	5.94	261
	Training	62 (77.5)	2,746	18.5	6.41	290
	*p*-value				0.7762	0.2260
Injury Category	Ligament	24 (30)	2,332	29	1.92	187
	Muscle	46 (57.5)	1,043	17.5	3.69	83.7
	Tendon	10 (12.5)	155	11	0.80	12.4
	*p*-value				2.6 × 10^−6^	0.0145
Severity	0–7 days	17 (21.2)	75	5	1.36	6.02
	8–28 days	38 (47.5)	663	17	3,05	53,2
	>28 days	25 (31.2)	2,792	54	2.01	224
	*p*-value				0.0151	2.6 × 10^−15^
Side	Left	38 (47.7)	1,883	20	3.05	151
	Right	42 (52.5)	1,647	21	3.37	132
	*p*-value				0.6546	0.9577

Total number of days lost is presented descriptively and not statistically compared between groups. Median days lost and injury burden are derived from the same comparison; therefore, a single *p*-value is reported.

A total of 80 injuries were recorded with an incidence of 6.42 per 1,000 h (95% CI: 5.09–7.99) and a burden of 283 days per 1,000 exposure hours (95% CI: 274–293). Eleven injuries (13.7%) occurred during menstrual bleeding phases and 69 (86.2%) during non-bleeding phases. Although the incidence of injuries was higher during the non-bleeding phase (6.48, 95%CI: 5.11–8.21) compared to the bleeding phase (5.36, 95% CI: 2.97–9.69) the difference was not statistically significant (*p* = 0.5519). However, the burden of injuries was significantly higher during bleeding phases (684, 95% CI: 649–722 vs. 206, 95%CI: 197–215; *p* = 0.0027), indicating a greater impact of injuries occurring during menstruation on total days lost.

When comparing injuries by physical activity context, 18 (22.5%) occurred during matches and 62 (77.5%), during training sessions. No significant differences were found in injury incidence (5.94 vs. 6.41; *p* = 0.7762) or burden (261vs. 290; *p* = 0.2260) between match and training contexts.

Regarding injury type ligament injuries represented 30% of all cases and had the highest burden (187 days lost) and the longest median day loss (29 days), with a significantly lower incidence compared to muscle injuries (1.92 vs. 3.69; *p* = 2.6 × 10^−6^). Muscle injuries were the most frequent (57.5%) but had a lower burden (83.7 days lost). Tendon injuries were less common (12.5%) and presented the lowest burden (12.4 days lost). The burden associated with ligament injuries was significantly higher compared to other injury types (*p* = 0.0145), as expected.

In terms of injury severity, 31.2% of injuries resulted in absences greater than 28 days. Injuries lasting 8–28 days accounted for the highest total day loss (47.5%, of the cases), while shorter injuries (0–7 days) comprised 21.2% of the sample, The incidence per 1,000 hours of exposure was 3.05 (8–28 days), 2.01 (+28 days) and 1.36 (0–7 days) and these observed differences in the incidence ratio were statistically significant (*p* = 0.0151).

Laterality analysis showed a near-even distribution between left (47.7%) and right (52.5%) side injuries, with no significant differences in incidence (3.05 vs. 3.37; *p* = 0.6546) or burden (151 vs. 132 days lost; *p* = 0.9577).

## Discussion

In this study, we aimed to accurately estimate the incidence and burden of injuries across the menstrual cycle using a large longitudinal design. Sport, and especially elite-level sport, is synonymous with tight schedules and frequent travel for competitions, which significantly complicates the ability to conduct studies involving the collection of biological samples. As highlighted in recent literature, accurately identifying distinct menstrual phases requires objective hormonal measurements, as hormone concentrations during the cycle are highly heterogeneous between individuals (22). Without these measurements, phase classification remains speculative (9), and the resulting incidence rates may be affected by measurement error, potentially influencing risk estimates and contributing to inconsistencies in the findings (11). Therefore, recognizing the potential limitations of our study, we sought to emphasize the value of the number of menstrual cycles recorded and its relationship with the injuries documented over the four competitive seasons, we focused on comparing injury incidence and burden between bleeding and non-bleeding phases, the only phases that can be reliably identified without hormone testing.

While previous studies have reported distinct incidence of injuries during the different phases of the menstrual cycle (3, 5, 7, 8), our large longitudinal study does not provide conclusive evidence of differences in overall injury incidence during menstrual bleeding. Although the incidence of injuries appeared lower during the bleeding phase, this difference was not statistically significant, likely due to wider confidence intervals and the smaller number of injuries recorded in this phase. Therefore, this observation should be interpreted with caution. The imbalance in exposure time— menstruation lasted an average of four days within a 31-day cycle, meaning that non-bleeding days accounted for approximately 27 days—more than six times the duration of the bleeding phase, may have limited the statistical power to detect differences in incidence. Nonetheless, the observed trends, combined with the significant difference in injury burden, underscore the importance of further research.

We found a significantly greater burden of injuries during the bleeding phase of the menstrual cycle, indicating that although injuries may not occur more frequently during this phase, they tend to be more severe and result in greater time loss. For example, we observed a more than threefold difference in the burden of soft tissue injuries (684 vs. 206 days lost per 1,000 h) during the bleeding phase compared to non-bleeding days among elite, menstruating female football players. This may be partly explained by the fact that two of the four cruciate ligament injuries occurred during the bleeding phase—injuries known to result in prolonged time loss from sport (23).

Although, available literature does not specifically demonstrate a difference in the severity of injuries based on menstrual cycle phase (24) it's known that the bleeding phase is characterized by low levels of oestrogen and progesterone, which may influence tissue properties and injury risk (25). Oestrogen exerts both protective and reparative effects on skeletal muscle, and its cyclical fluctuations throughout the menstrual cycle have been shown to influence the risk, severity, and recovery of musculoskeletal injuries in menstruating individuals (26). Several studies have reported that the early follicular phase—characterized by low circulating oestrogen levels—is associated with increased susceptibility to exercise-induced muscle damage, heightened delayed onset muscle soreness (DOMS), and greater post-injury strength loss. These findings suggest a phase-dependent vulnerability and potentially slower recovery during periods of low oestrogen availability (25, 27). Therefore, our results, underscore the need for larger studies with precise menstrual data, including hormonal assessments, and the importance of accounting for additional factors such as internal and external training loads (28, 29).

Our study, however, suggest that the substantial increase in disruptive injuries, reflected by the higher injury burden, during the bleeding phase may not be solely explained by the low levels of steroid hormones typically present during this phase. This finding contrasts with the commonly hypothesized susceptibility to non-contact injuries associated with high hormone levels (such as oestrogen), which have been linked to decreased tissue stiffness and impaired neuromuscular control (30, 31). It is also important to consider that hormone levels during the proliferative and luteal phases vary considerably between individuals. For example, some athletes show progesterone concentrations below expected thresholds (32), indicating possible subclinical ovulatory disturbances, ranging from anovulation to insufficient progesterone production throughout the cycle. Therefore, menstruation bleeding may not always serve as a reliable proxy for a period of consistently ovarian hormonal concentrations.

While understanding individual hormonal fluctuations is essential—given that injury risk in female athletes is multifactorial (28)—non-hormonal factors during the bleeding phase, such as training load, fatigue, symptom severity, nutrition, and recovery, likely play a substantial role in injury severity (33–36). Supporting our findings, a recent study using symptom tracking and surveys in recreationally active women reported that symptom severity peaked during menstruation and was associated with perceived declines in performance and longer recovery times (30), both of which may contribute to a higher risk of severe injuries. The inclusion of these additional factors is therefore necessary to improve the interpretability and reliability of future research findings (37).

## Limitations

The unequal distribution of days between bleeding and non-bleeding phases limits the statistical power to accurately estimate injury risk during menstrual bleeding. Additionally, other important factors known to influence injury risk, such as sleep quality, nutrition, and symptom severity, were not assessed in this study. Moreover, the study spanned four different seasons, including the 2019/20 season, which was affected by the COVID-19 pandemic, as well as periods of coaching changes and player turnover. These factors may have introduced heterogeneity within the sample.

Despite these limitations, our four-season study has several important strengths. All athletes belonged to the same club, followed consistent injury prevention protocols, had access to professional medical support, and trained under standardized methods. These factors helped reduce variability and strengthen the reliability of our findings.

### Practical applications

A calendar-based method can be used as a practical tool to collect health information and help identify the risk of disruptive injuries. It is an affordable approach that can complement other measurements in injury prediction models.

## Conclusions

This four-season longitudinal study in elite female football players found that while the overall incidence of injuries was similar between bleeding and non-bleeding phases of the menstrual cycle, injuries occurring during menstruation were associated with significantly greater severity, as reflected by a higher injury burden. These findings suggest that menstruation may not increase the likelihood of injury but could be linked to more disruptive outcomes when injuries occur. The study highlights the importance of individualized menstrual tracking and the consideration of both hormonal and non-hormonal factors—such as training load, fatigue, and symptom severity—in injury prevention strategies. Future research should incorporate objective hormonal measurements and broader physiological and contextual variables to improve the understanding and management of injury risk in female athletes.

## Data Availability

The original contributions presented in the study are included in the article/Supplementary Material, further inquiries can be directed to the corresponding author.
